# IoT-Enabled Wireless Sensor Networks for Air Pollution Monitoring with Extended Fractional-Order Kalman Filtering

**DOI:** 10.3390/s21165313

**Published:** 2021-08-06

**Authors:** Santanu Metia, Huynh A. D. Nguyen, Quang Phuc Ha

**Affiliations:** 1 Faculty of Engineering and Information Technology, University of Technology Sydney, Sydney, NSW 2007, Australia; Santanu.Metia@uts.edu.au (S.M.); Huynhanhduy.Nguyen@student.uts.edu.au (H.A.D.N.); 2 College of Engineering Technology, Can Tho University, Can Tho 900000, Vietnam

**Keywords:** extended fractional-order kalman filter, internet of things, air quality

## Abstract

This paper presents the development of high-performance wireless sensor networks for local monitoring of air pollution. The proposed system, enabled by the Internet of Things (IoT), is based on low-cost sensors collocated in a redundant configuration for collecting and transferring air quality data. Reliability and accuracy of the monitoring system are enhanced by using extended fractional-order Kalman filtering (EFKF) for data assimilation and recovery of the missing information. Its effectiveness is verified through monitoring particulate matters at a suburban site during the wildfire season 2019–2020 and the Coronavirus disease 2019 (COVID-19) lockdown period. The proposed approach is of interest to achieve microclimate responsiveness in a local area.

## 1. Introduction

According to the World Health Organization, air pollutants were responsible for approximately 58% of total deaths related to cardiovascular problems, with nearly one-fifth of the cases being from serious lung illnesses (e.g., obstructive pulmonary or respiratory infections), and lung cancer constituted 6% of all mortalities in 2016 [1]. Poor air quality, especially regarding excessive particulate matter (PM), has an impact not only on living beings, but also on ecosystems [2,3] and the Earth’s climate [4,5].

The requirement for accurate monitoring and forecasting systems for real-time air quality is one of the top priorities in metropolitan areas. Notably, due to the recent proliferation of the Internet of Things (IoT) technologies, low-cost wireless sensor networks (LWSN) have been designed and have developed rapidly as local monitoring systems in conjunction with the state-run observatories to enhance the spatio-temporal distribution resolution of environmental parameters [6,7]. However, as these sensors are often exposed to changes of weather patterns in their 24/7 operation, it is essential to develop reliable and resilient monitoring systems for performance improvement.

Reliability and quality of the wireless monitoring systems remain an ongoing research topic which has attracted researchers from different disciplines such as as smart calibration [8], probability models [9,10], or clustering adaptations (e.g., LEACH clustering protocols and fuzzy) [11,12]. However, the limited resources for low-cost, low-power WSN remains an obstacle for the real-time application of these techniques, of which most results are obtained in simulation. Therefore, the realization of large-scale implementation for an air quality monitoring system based on IoT platforms is interesting to explore.

For air quality monitoring, a number of heterogeneous sensors are used in each sensor mote to measure several to dozens of parameters, such as particulate matter, ozone, sulfur oxides, volatile oxide compounds, ammonia, carbon oxides, nitrogen oxides, and other meteorological variables. The number of sensor motes are also densely located over the study area for comprehensive coverage and high spatio-temporal resolution of the collected data. Therefore, the fusion of these wireless sensing data is required for enhancing performance of the monitoring system [13,14].

In LWSN, data assimilation is of crucial importance, for which preconditioning and filtering techniques have been used in the first stage. To deal with imperfect conditions such as missing information, packet losses and stochastic uncertainty, and sensor noise associated with the time series of air quality data collected, Kalman filtering approaches have been proposed with a basic form [15] and various types of Kalman filters. An accuracy improvement is obtained both in monitoring and prediction of different air pollutants by using Kalman filtering compared with common forecasting models [16]. An adaptive Kalman filter based on an autoregressive model is proposed for sensing and prediction of Air Quality Index values in [17]. Unscented Kalman filters are developed for short-term wind speed prediction [18], and for urban air pollution prediction with scaled monitoring data [19].

This paper presents the design and implementation of an IoT-enabled LWSN with enhanced accuracy and reliability for monitoring local air quality. The development is applied for a suburban construction site to monitor emissions of particulate matters. Here, a wireless dependable control (W-DepC) scheme is integrated with extended fractional-order Kalman filtering (EFKF) for data assimilation and imputation to deal with missing information and climate volatility to enhance the system performance. The proposed system offers a reliable and accurate solution to the problem of air quality monitoring for microclimate responsiveness, which is the main contribution of this paper. On-field results are obtained and analyzed to demonstrate the effectiveness of this approach in local air pollution prediction.

The paper is organized as follows. After the introduction, the proposed LWSN system and dependable schemes based on redundancy configurations are presented in Section 2. Section 3 describes the EFKF algorithm developed for data collection and recovery the missing information. The real-world system for monitoring dust emissions from a construction site is reported in Section 4. A discussion on the results obtained from the proposed system using the proposed EFKF is given in Section 5. Finally, a conclusion and future work are given in Section 6.

## 2. IoT-Enabled LWSN System Design

This section introduces the design of the proposed IoT-enabled wireless low-cost sensor network: its system description, dependable monitoring schemes, and laboratorial validation.

### 2.1. System Description

To enhance the network interfacing and communication capacity of the LWSN, IoT technology is used to enable the communication and exchange of information within and between each sensor mote. As the cost of IoT components has been significantly reduced, the number of applications of IoT technology is increasing in many smart devices and systems. In environmental monitoring, this inexpensive solution also contributes to ease the problem of system failures under the adverse impacts of weather events in a local area by improving accuracy and reliability.

**Figure 1:**
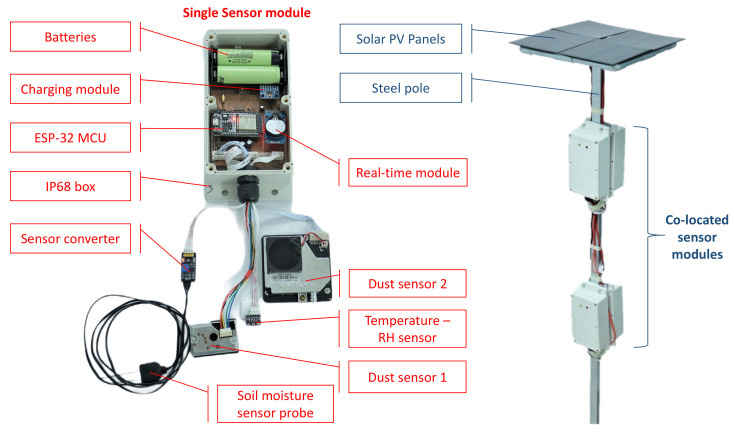
Prototype of a sensor module (**left**) connected with multiple low-cost sensors and a sensor mote (**right**) including four collocated sensor modules attached on the steel pole with 4 mini solar PV panels.

For the proposed LWSN, two IoT modules are developed including the sensor mote for remote data collection and the router mote (gateway) for data relaying and framework controlling. In this paper, a sensor mote comprises a number of sensor modules in a redundant configuration. A module operates independently with other modules and exchanges information in the layer of a mote, while a mote communicates with other motes over the whole network. Figure 1 shows a sensor mote comprising four sensor modules, each contains one micro-control unit (MCU), environmental sensors (soil moisture, soil temperature, relative humidity (RH), pressure, particulate matters (PM2.5 and PM10), two battery cells (Panasonic Lithium-Ion 18,650, 3.7V-3400 mAh/cell), one mini solar PV panel, a real-time reading unit, boost-buck converter, and battery chargers. Specifications of components used and current costs are typically given in Table 1.

### 2.2. Dependable Monitoring Scheme

**Figure 2:**
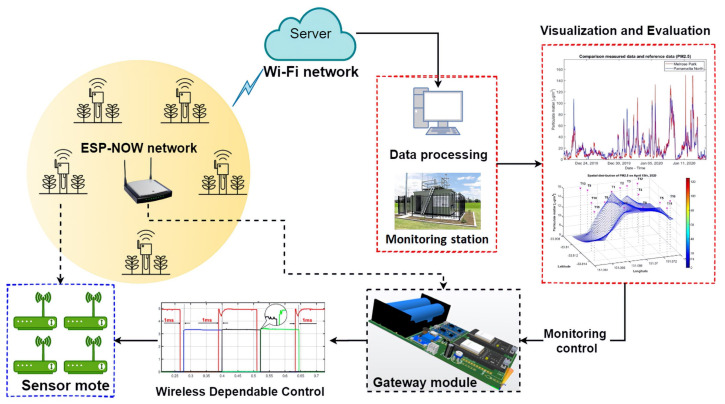
W-DepC framework with co-located LWSN.

To cope with environmental volatility, air quality monitoring systems must be reliable and resilient. These properties of a dynamic system are collectively known as dependability, which covers availability, reliability, maintainability, durability, and security [20]. Regarding environmental volatility, our proposed IoT-enabled system applies partially this standard to enhance IoT system reliability and data quality by a strategy called dependable monitoring scheme. This scheme is attributed to a redundant strategy with four co-located sensor modules in each sensor mote (quad mode), as presented in Figure 2. The sensor modules communicate with the gateway in a local network while the raw data are transmitted via Wi-Fi to the cloud server. This ESP-NOW network is a low-power wireless communication protocol featuring the ESP32 device and MCU. Then, the collected data are processed and validated by means of visualization and statistical calculation in comparison with benchmarking data from the state-run monitoring systems.

In this work, the mote gateway is configured with two ESP32 IoT modules connected to each other by the serial communication protocol (RS232). One IoT-module communicates regularly with other sensor motes by the local network (ESP-NOW), wherein the W-DepC is applied for dependable monitoring. Therefore, not only interference between two communication protocols is avoidable, but system reliability can also be improved. The remaining IoT-MCU is named as global gateway to play the role of transmitting data to the cloud (a Thingspeak IoT server). As illustrated in Figure 2, the yellow circle is a local network including one gateway module communicating with neighboring sensor motes that are, in turn, controlled by the W-DepC. The outer space of the local network is a global network (Wi-Fi network) used to transmit the raw environmental data to the server for later processing and evaluating.

All the main components and printed circuit boards are protected in waterproof enclosures (IP68 standard) from intrusive water and on-site dust. Although each sensor module is designed separately, four modules are arranged on the same location, each controlled under the proposed W-DepC schemes, ensuring dependability of the sensor mote. Descriptions of the prototypical sensor module with its components and the sensor mote of quad-modules, as shown in Figure 1, as well as the energy conservation, calibration, and performance testing in laboratorial conditions of this system are included in [21].

The monitoring process is controlled and updated to the gateway regularly to manage the operation of sensor modules through W-DepC scheme [22], whereby a so-called “switching” mechanism is designed to minimize a quadratic function based on its energy state to select the most reliable sensor module in each sensor mote [21]. At an instant, this mechanism triggers only one active module. The on-duty sensor module can communicate wirelessly with gateways while the others are in the stand-by mode, i.e., staying off-line but still collecting the on-site data to conserve battery energy and assure data continuity. Beside the physical redundancy, the network coverage is also divided into two layers: (i) the local layer with ESP-NOW protocol between local gateways and sensor motes, and (ii) the global layer with Wi-Fi 802.11 protocol. This arrangement enhances reliable connection for a high number of modules and assures the continuous operation of the developed LWSN.

### 2.3. Test Validation

To maintain the service especially in remote operation, LWSN have to deal with the power-constrained problem. Here, the W-DepC mitigates the number of active sensor modules in each mote and controls the sampling frequencies. The calculation and validation experiment of the draining energy during active and deep-sleep modes of each sensor module are reported in [21]. As normal sampling periods in air pollution domain are relatively slow (minutes or hours), the programmable sampling periods rely on the sleeping intervals to reduce the energy waste of temporal redundancy of the collected data. Here, the dust sensors require a stable time for reading ($T_{stable}$), which is fixed at 30 s. It is then augmented with the sleeping interval ($T_{sleep}$) and the reading interval ($T_{read}$) to yield the sampling period $T_{sampling}=T_{sleep}+T_{stable}+T_{read}$ for a sampling frequency of up to 6 kHz as per the datasheet [23]. With the integration of a solar PV panel, the proposed system can operate continuously at sampling frequencies less than 1 Hz. Taking account of the weather extreme events, the sampling period is selected at 15 min.

## 3. Extended Fractional-Order Kalman Filtering (EFKF) Algorithm

To deal with nonlinearity in dynamical systems, the extended Kalman filter (EKF) is often used for estimation of system states. In EKF applications, the measurements from sensors are assumed free of such issues such as missing data, packet losses, or additive uncertainty in the gain [24]. However, for sensor networks, especially IoT-enabled wireless sensor networks, missing measurements [25] and packet dropouts [26] occur quite often in practice. Moreover, inevitable dropouts of LWSN data are often encountered during transmission process from sensor motes to the server. Therefore, it is important to seek an EKF-based variant to improve the accuracy of the estimation in these monitoring systems. To this end, the extended fractional-order Kalman filter (EFKF) has appeared to be promising in many applications in industry [27]. With EFKF, the accuracy of the estimation can be improved via better modeling the dynamic system, smoothing out measurement noise from low-cost sensors and recovery of missing information. For estimation of air pollutant emissions, EFKF has been used as an approach to the inverse modeling [28] or to improve the air pollution prediction from the emissions inventory [29]. In the following, after a brief description on the EFKF derivation, its algorithm used for processing data collected from the LWSN for monitoring local air quality is introduced in this section.

### 3.1. EFKF Derivation

Let us first consider a fractional derivative defined by Grünwald–Letnikov as

(1) $$\begin{array}{c} \Delta^{\alpha }x_{k}=\frac{1}{h^{\alpha }}\sum_{j=0}^{k}{\left(-1\right)}^{j}\left(\begin{array}{c} \alpha \\ j \end{array}\right)x_{k-j}, \end{array}$$

where *h* is the step size, α is the order of fractional difference, *k* is the number of samples, and *j* is an integer index starting from 0 to *k*, in the following factor:

(2) $$\begin{array}{c} \left(\begin{array}{c} \alpha \\ j \end{array}\right)=\left\{\begin{array}{cc} 1 & \mathrm{if}\;j=0,\\ \frac{\alpha (\alpha -1)\cdots (\alpha -j+1)}{j!} & \mathrm{if}\;j>0. \end{array}\right. \end{array}$$

Then, the discretized model of a dynamic system can be expressed in the stochastic space [24,27]. It is expressed as

(3) $$\begin{array}{c} \Delta^{\alpha }x_{k+1}=f\left(x_{k},u_{k}\right)+w_{k}, \end{array}$$

(4) $$\begin{array}{c} x_{k+1}=\Delta^{\alpha }x_{k+1}-\sum_{j=1}^{k+1}{\left(-1\right)}^{j}\Upsilon_{j}x_{k+1-j}, \end{array}$$

(5) $$\begin{array}{c} y_{k}=g\left(x_{k}\right)+v_{k}, \end{array}$$

where f(·) and g(·) are nonlinear functions, $x_{k}$ is the state vector, $u_{k}$ is control input, $y_{k}$ is measurement output, $w_{k}$ is the system noise at time instant *k*, and $v_{k}$ is the measurement noise at time instant *k*. Both $w_{k}$ and $v_{k}$ are considered Gaussian noise. The covariance matrices of $w_{k}$ and $v_{k}$ are given by

(6) $$\begin{array}{c} E\left[\begin{array}{c} w_{i}w_{j}^{T} \end{array}\right]=\left\{\begin{array}{cc} Q_{i} & \mathrm{if}\;i=j,\\ 0 & \mathrm{if}\;i\neq j, \end{array}\right. \end{array}$$

(7) $$\begin{array}{c} E\left[\begin{array}{c} v_{i}v_{j}^{T} \end{array}\right]=\left\{\begin{array}{cc} R_{i} & \mathrm{if}\;i=j,\\ 0 & \mathrm{if}\;i\neq j, \end{array}\right. \end{array}$$

and

(8) $$\begin{array}{c} \Upsilon_{j}=\mathrm{diag}\left(\begin{array}{c} \left[\begin{array}{c} \left(\begin{array}{c} \alpha_{1}\\ j \end{array}\right),\left(\begin{array}{c} \alpha_{2}\\ j \end{array}\right),\cdots ,\left(\begin{array}{c} \alpha_{N}\\ j \end{array}\right) \end{array}\right] \end{array}\right), \end{array}$$

where $Q_{i}$ and $R_{i}$ are the covariance matrices of system noise and measurement noise, respectively, at time instant *i*; $\alpha_{1},\alpha_{2},\cdots ,\alpha_{N}$ are the orders of system equations; *N* is the number of these equations; and j=1,2,⋯,k+1.

The estimation of the system state vector is obtained *a priori* from minimizing the cost function [24,28]:

(9) $$\begin{array}{c} \hat{x_{k}}=\arg_{x_{k}}\min\left[\begin{array}{c} \left[\begin{array}{c} \tilde{x_{k}}-x_{k} \end{array}\right]{\tilde{P}}_{k}^{-1}{\left[\begin{array}{c} \tilde{x_{k}}-x_{k} \end{array}\right]}^{T}+\left[\begin{array}{c} y_{k}-g\left(x_{k}\right) \end{array}\right]R_{k}^{-1}{\left[\begin{array}{c} y_{k}-g\left(x_{k}\right) \end{array}\right]}^{T} \end{array}\right], \end{array}$$

where

(10) $$\begin{array}{c} \tilde{x_{k}}=E\left[x_{k}|y_{k-1}^{*}\right] \end{array}$$

is the prediction vector of the system state at time instant *k* given the measurement sequence at (k−1), $y_{k-1}^{*}$ containing values of the input $u_{0},u_{1},\cdots ,u_{k-1}$ and output $y_{0},y_{1},\cdots ,y_{k-1}$. The estimation vector of the system state updated *a posteriori* at time instant *k* by the EFKF is

(11) $$\begin{array}{c} {\hat{x}}_{k|k}=E\left[x_{k}|y_{k}^{*}\right]. \end{array}$$

Here, the covariance matrix of prediction error ${\tilde{P}}_{k}$ and the covariance matrix of estimation error ${\tilde{P}}_{k}$ at time instant *k* are, respectively,

(12) $$\begin{array}{c} {\tilde{P}}_{k}=E\left[\left(x_{k}-{\tilde{x}}_{k}\right){\left(x_{k}-{\tilde{x}}_{k}\right)}^{T}\right], \end{array}$$

and

(13) $$\begin{array}{c} {\hat{P}}_{k}=E\left[\left(x_{k}-{\hat{x}}_{k}\right){\left(x_{k}-{\hat{x}}_{k}\right)}^{T}\right]. \end{array}$$

### 3.2. EFKF Implementation

To implement data assimilation for the proposed LWSN, the following standard extended Kalman filtering Algorithm 1 is applied for the data collected $y_{k-1}^{*}$ from the LWSN and the concentrations of the air pollutants of interest $y_{k}$ in association with the emissions process described by Equations (3)–(5). Notably, if the fractional-order derivative in Equations (3) and (4) is replaced by a conventional derivative, then the algorithm will be the extended Kalman filter (EKF).

In terms of software packages for fractional-order systems, there are some fractional-order model (FOM) identification toolboxes available, such as CRONE toolbox [30] and FOMCON toolbox [31]. They are based on Hartley [32] and Levy [33] algorithms to obtain a commensurate fractional-order transfer function (FOT) for a given system. The FOMCON toolbox has fractional-order analysis module, identification (time and frequency) module, control (fractional order proportional integral derivative (FOPID)) module, and implementation (digital and analog fractional order (FO)) module. Considering those advantages, we used the FOMCON toolbox to identify fractional orders in the time domain. Details of the application for the study area and different scenarios (bushfires, normal, and COVID-19) are mentioned in the next section.
**Algorithm 1** Standard extended Kalman filtering algorithm1:Set: Intial $\hat{x_{0}}$ and $\hat{P_{0}}$
$$\begin{array}{c} \hat{x_{0}}=E\left[\begin{array}{c} x(0) \end{array}\right]=x_{0}, \end{array}$$
$$\begin{array}{c} \hat{P_{0}}=E\left[\begin{array}{c} \left(x\left(0\right)-x_{0}\right){\left(x\left(0\right)-x_{0}\right)}^{T} \end{array}\right]=P_{x_{0}}, \end{array}$$2:Compute:
$$\begin{array}{c} F_{k-1}=\frac{\partial f(x_{k-1},u_{k-1})}{\partial x_{k-1}}{|}_{x_{k-1}={\hat{x}}_{k-1}}, \end{array}$$3:**if**k≤L**then**4:       Predict:
$$\begin{array}{c} \tilde{x_{k}}=f\left({\hat{x}}_{k-1},u_{k-1}\right)-\sum_{j=1}^{k}{\left(-1\right)}^{j}\Upsilon_{j}{\hat{x}}_{k-j}, \end{array}$$
$$\begin{array}{c} \tilde{P_{k}}=\left(F_{k-1}+\Upsilon_{1}\right){\hat{P}}_{k-1}{\left(F_{k-1}+\Upsilon_{1}\right)}^{T}+Q_{k-1}+\sum_{j=2}^{k}\Upsilon_{j}{\hat{P}}_{k-j}\Upsilon_{j}^{T}, \end{array}$$5:       Compute:
$$\begin{array}{c} H_{k}=\frac{\partial g(x_{k})}{\partial x_{k}}{|}_{x_{k}=\tilde{x_{k}}}, \end{array}$$
$$\begin{array}{c} K_{k}=\tilde{P_{k}}H_{k}^{T}{\left(H_{k}\tilde{P_{k}}H_{k}^{T}+R_{k}\right)}^{-1}, \end{array}$$6:       Update:
$$\begin{array}{c} \hat{x_{k}}=\tilde{x_{k}}+K_{k}\left[y_{k}-g\left(\tilde{x_{k}}\right)\right], \end{array}$$
$$\begin{array}{c} \hat{P_{k}}=\left(I-K_{k}H_{k}\right)\tilde{P_{k}}, \end{array}$$7:**else**8:      k>L9:      Stop10:**end if**      where *L* is the length in consideration.

## 4. Suburban Air Quality Monitoring System

To collect air quality data for the State of New South Wales (NSW), Australia, there are nearly one-hundred State-run monitoring stations across the whole state and about 20 active stations in the Sydney basin. They are under the management of Department of Planning, Industry and Environment (DPIE) of the NSW Government. These stations provide benchmarking data, which are freely accessible and downloadable from a database of hourly and daily values of air pollutants and meteorological variables [34]. As the number of monitoring stations is limited, LWSN offers a promising solution to the problem of local air quality monitoring in the suburbs.

### 4.1. Description of the Study Area and Experiment Period

**Figure 3:**
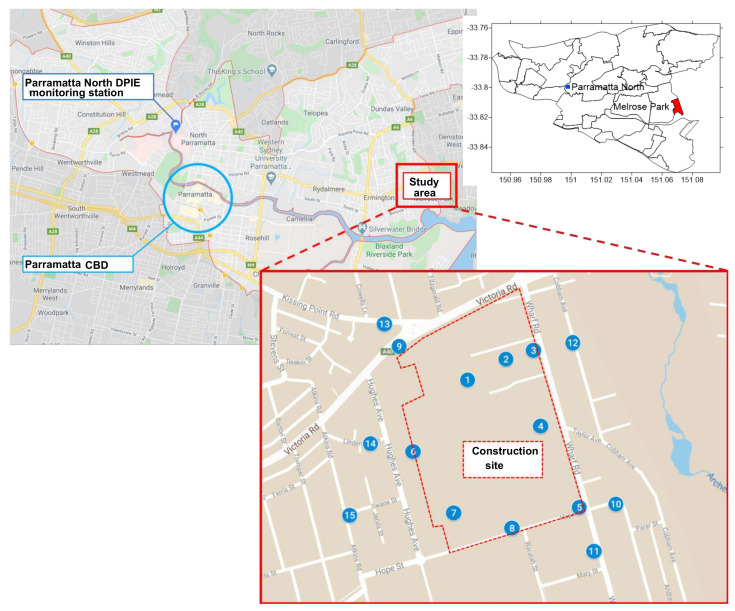
Map showing EMS deployments relatively to a closest monitoring station.

In this work, the proposed monitoring system is applied to monitor the emissions of particulate matter from a construction site in Melrose Park (MP), a suburb located between Sydney Central-East and Sydney North-West regions, to which the closest state-run station is at Parramatta North (PN). The suburb is being developed as a major new mixed residential, business, and retail precinct over the next ten years. Active development at the site commenced from the second half of 2019. Figure 3 depicts the study area, where 15 sensor motes are installed at the site as well as its surrounding residential area at MP to monitor the air pollution levels contributed from construction activities. Here, the pollutants of concern are dust, namely, PM2.5 and PM10 concentrations. The sensor motes use the commercial Environment Monitoring System (EMS), which can measure the levels of different air pollutants. The raw data are extracted among the EMS group to collect multiple air quality parameters, including temperature; relative humidity; carbon monoxide; nitrogen dioxide; ozone; and particulate matters PM1.0, PM2.5, and PM10. The study period is from 15 November 2019 to 7 May 2020, in coincidence with a devastating wildfire disaster in NSW and the COVID-19 pandemic. The measurements are sampled at a period of 15 min, which assures the stable operation of the sensors, network connection, and battery’s function due to the slow variations of ambient variables. Concentrations of PM2.5, PM10, and PM1.0 are expressed in micrograms of particles per cubic meter of air (μg/m${}^{3}$).

**Figure 4:**
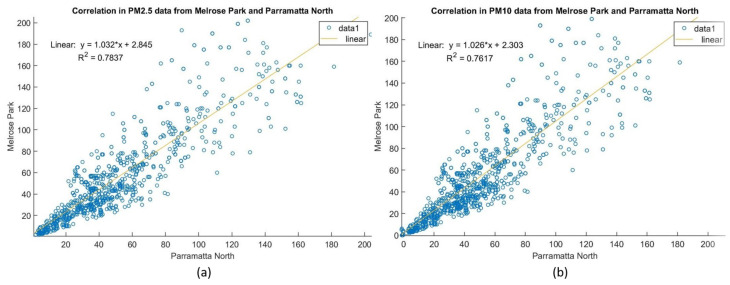
Scatter plots: (**a**) PM2.5 and (**b**) PM10 collected at the site and the station.

For validation of the IoT-enabled monitoring system, the measured data from the LWSN were compared with those collected at the closest State-run monitoring station and then properly processed in order to avoid missing information or abnormalities from imperfect conditions [35]. By linear regression, an agreement between the measured data at MP and the reference data at the monitoring station at PN via the correlations, respectively, for PM2.5 and PM10, is shown in Figure 4. The fitting lines with calculated coefficient of determination $R^{2}$ (0.7837 for PM2.5 as opposed to 0.7617 for PM10) represent the linear relationship of the two datasets with a slope greater than one, implying emissions at MP were more serious than at PN in those days (possibly from the construction activities). Furthermore, by comparing the two linear regression slopes, it seems that emissions of fine particles PM2.5 at MP may affect the surrounding environment slightly more than particulate matters PM10.

Notably, the study period covers three stages. In the first period from November 2019 to January 2020, there was a surge in the levels of particulate matters due to a catastrophic bushfire event in NSW. For the latter period from March to May 2020, due to the coronavirus pandemic the whole NSW state was also placed under lockdown, and thus transport emissions were significantly reduced. Between the bushfire and the lockdown periods, the month February 2020 can be considered as normal.

### 4.2. Bushfire Period

**Figure 5:**
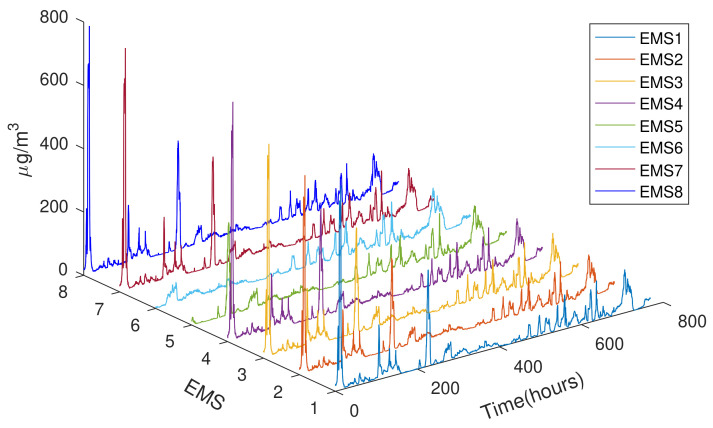
On-site PM2.5 concentration during 10 DEC 2019–10 JAN 2020.

During the Black Summer 2019–2020, the Sydney area was affected by extremely high concentrations of particulate matters in the air as a result of severe bushfires. Figure 5 shows the temporal distributions of PM2.5 using real-time data collected by eight sensor motes, EMS1–8, of the LWSN from 10 December 2019 to 10 January 2020.

**Figure 6:**
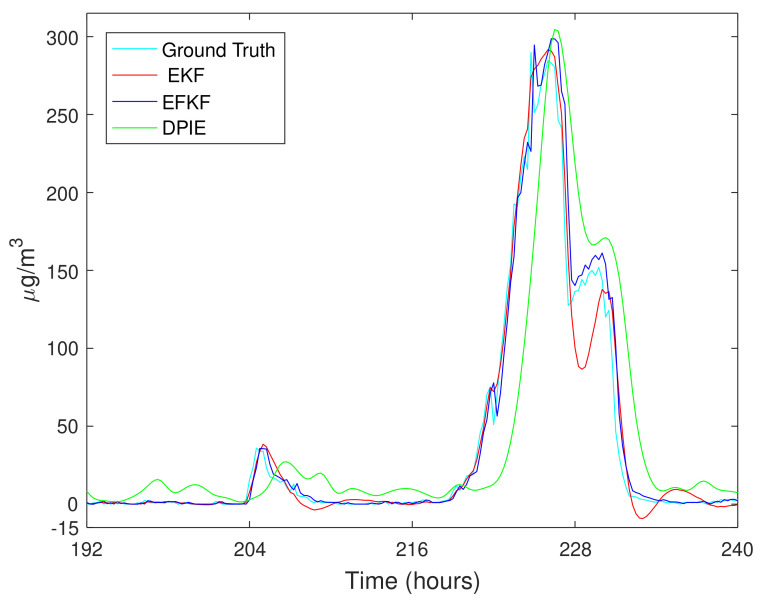
On-site PM2.5 concentration during 18–19 December 2019: ground truth (measured data), DPIE (station data), and estimation using EKF and EFKF.

In Figure 6, local monitoring data collected in real-time from EMS2 of the LWSN, EKF- and EFKF-processed estimations, and referencing data obtained from a closest state-run monitoring station are plotted for the PM2.5 concentration in two days, 18–19 December 2019. These data clearly indicate that the EFKF can reflect accurately any variations in local air pollutant profiles as compared to the LWSN measurements for air pollution monitoring at a local suburb.

**Figure 7:**
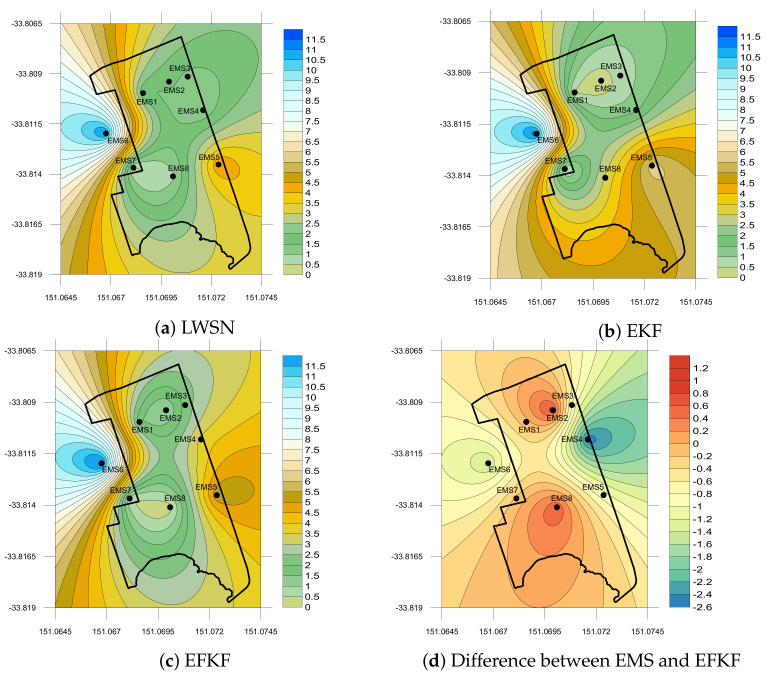
Local PM2.5 (μg/m${}^{3}$) concentration on 19 December 2019 at 0:15 a.m.

The spatial plots of fine dust PM2.5 concentrations distributed at the suburb site are shown in Figure 7 using data from EMS measurement, EKF and EFKF estimations, and the difference between EMS and EFKF data on 19 December 2019. It can be observed of low emissions of PM2.5 at midnight (0:15 a.m.). With a small discrepancy of less than 1.2 μg/m${}^{3}$ as compared to data from the sensor motes, the EFKF can be used to provide accurate prediction of air pollutant profiles for suburban air quality monitoring.

### 4.3. Normal Session

**Figure 8:**
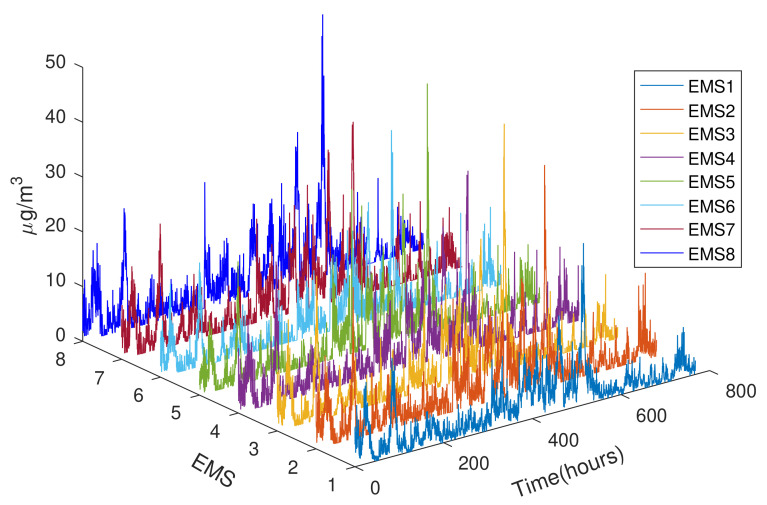
On-site PM10 concentration during 15 February–17 March 2020.

At the end of Summer 2020 when all bushfires were extinguished across NSW, air quality can be considered normal. During the period from 15 February 2020 to 17 March 2020, the hourly variations of particles PM10 are plotted in Figure 8, profiling air quality data collected by the eight EMS sensor motes, EMS1–EMS8. They all show a level of dust emissions less than 50 μg/m${}^{3}$, indicating good air quality given construction activities and heavy traffic at the site of interest.

**Figure 9:**
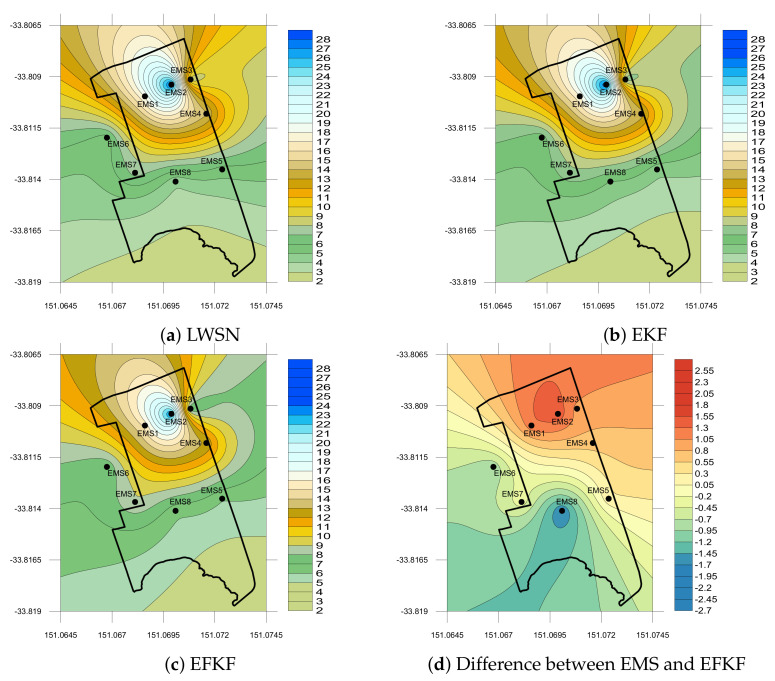
Local PM10 (μg/m${}^{3}$) concentration on 18 FEB 2020 at 11:45 am.

Spatial plots are shown in Figure 9 for EMS data from the LWSN, EKF and EFKF estimated distributions, and the difference between EMS and EFKF profiles of PM10 emissions on 18February 2020 at midday. The results obtained confirm the merit of the proposed LWSN with EFKF, with a difference between the EMS measurements and EFKF estimations distributed over the site of interest that is mostly smaller than 2 μg/m${}^{3}$ over the site with the most accurate area being located in the middle and some under estimation observed at the locations of EMS1, EMS2, and EMS3 perhaps due to construction activities.

### 4.4. COVID-19 Session

The period from 3 March 2020 to 4 April 2020 was coincident with the COVID-19 lockdown, when emissions from transportation emissions were significantly reduced. To demonstrate the capability of the sensor motes of our LWSN for local monitoring of finer particles, we consider PM1 concentrations distributed over the study area at 7:00 am on 3 April 2020.

**Figure 10:**
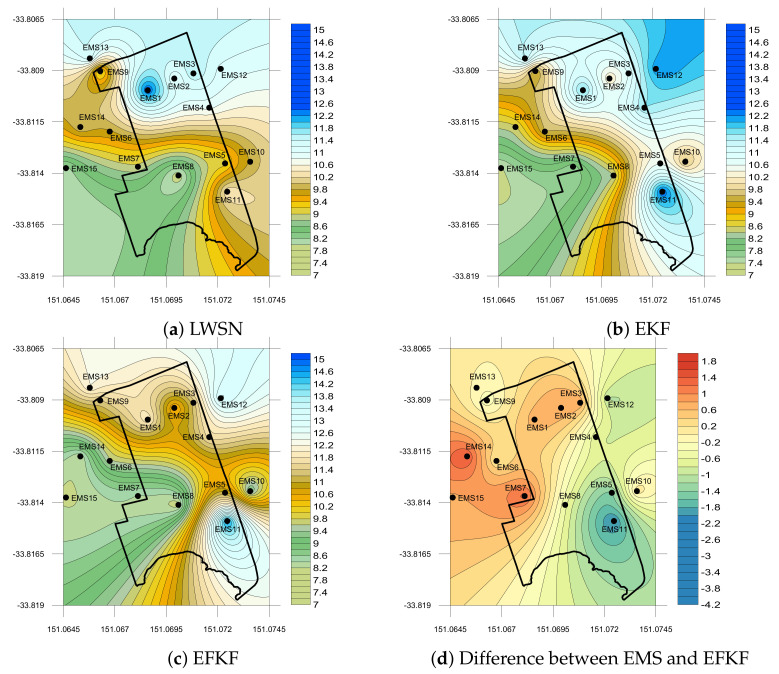
Local PM1 (μg/m${}^{3}$) concentration on 3 APR 2020 at 7:00 am.

Spatial plots of data from 15 sensor motes EMS1-EMS15, estimations from both EKF and EFKF, and the difference in PM1 concentrations between LWSN measurements and EKKF distributions are depicted in Figure 10. Again, the EFKF used for the proposed low-cost monitoring system can provide accurate estimation of the pollutant PM1 profiles with an error of the spatial distributions of less than 1 μg/m${}^{3}$ over a small-scale suburban site.

## 5. Discussion

**Figure 11:**
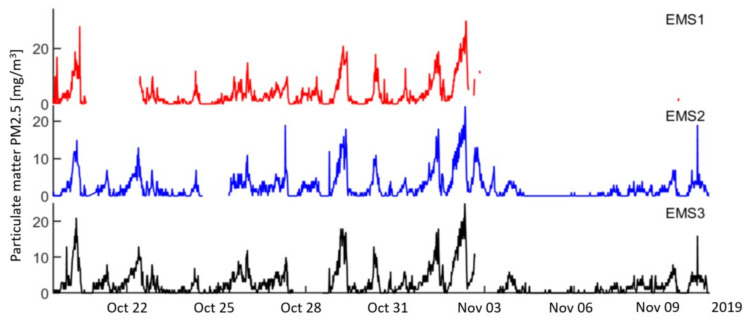
Data collected at three co-located sensor modules: EMS1, EMS2, and EMS3.

The results obtained indicate that the proposed LWSN with EFKF for monitoring of air quality data from low-cost sensors offers a great alternative to air quality monitoring in local suburbs, where State-run stations can hardly cover all locations or provide a comprehensive interpretation with visualization and dashboard. Here, the IoT-enabled architecture incorporating a dependable collocated network can allow for high-resolution spatio-temporal pollutant profiles and pollution exposure maps for local areas with improved accuracy. As mentioned above, handling data imperfections in wireless sensor networks remains a common problem for which the EFKF algorithm is used in this work. To illustrate this, we consider the monitoring data collected from three sensor motes—EMS1, EMS2, and EMS3—over a 25-day period from 19 October to 12 November 2019. Regarding fine particles PM2.5, the measurements from the sensor motes are plotted in Figure 11, where a strong correlation ($R^{2}\geq 0.9$) is obtained for the temporal patterns. However, missing values can be observed at random time instant as well as in various periods for all the sensors. Here, the EFKF algorithm is utilized for the LWSN monitoring system as a remedy for this unavoidable issue.

To further evaluate the system merits, the performance metrics used here include the mean absolute error (MAE), root mean squared error (RMSE), and Pearson correlation coefficient (PCC), also referred to as Pearson’s *r*:

(14) $$\begin{array}{c} \mathrm{MAE}=\frac{\sum_{i=1}^{n}\left|y_{i}-x_{i}\right|}{n}, \end{array}$$

(15) $$\begin{array}{c} \mathrm{RMSE}=\sqrt{\frac{\sum_{i=1}^{n}{\left(y_{i}-x_{i}\right)}^{2}}{n}}, \end{array}$$

(16) $$\begin{array}{c} \mathrm{PCC}=r_{xy}=\frac{\sum_{i=1}^{n}\left(x_{i}-\bar{x}\right)\left(y_{i}-\bar{y}\right)}{\sqrt{\sum_{i=1}^{n}{\left(x_{i}-\bar{x}\right)}^{2}}\sqrt{\sum_{i=1}^{n}{\left(y_{i}-\bar{y}\right)}^{2}}}, \end{array}$$

where $y_{i}$ is the prediction, $x_{i}$ is the true value, *n* refers to the total number of data points, the sample mean is $\bar{x}=\frac{\sum_{i=1}^{n}x_{i}}{n}$, and the prediction mean is $\bar{y}=\frac{\sum_{i=1}^{n}y_{i}}{n}$.

Table 2 summarizes performance statistics for PM2.5 particulate matter during the Back Summer bushfire. From the tabulated MAE and RMSE, it is obvious that the fractional derivative in Equations (3) and (4) represents a more accurate model for the emissions process resulting in an increase in accuracy of about 2–3 times for EFKF compared to EKF. The Pearson’s correlation coefficient is closer to 1, indicating a better positive linear relationship between the estimations and the sensors network data. Similar results are expected for coarse particles PM10. Table 3 displays the MAE, RMSE, and PCC calculated for eight sensor motes EMS1–EMS8 of the LWSN during a normal session. During the COVID-19 period, the 15 sensor motes EMS1–EMS15 were in operation. The same statistical characteristics of the system are given in Table 4. We can see that in terms of MAE and RMSE, the prediction with the EFKF model is approximately 3–4 times more accurate than with EKF. The system is promising for a local neighborhood to become climate-responsive in face of weather volatility, which is currently an important aspect in moving towards smart cities.

Given a number of existing air quality models and the availability of other information sources, such as population, satellite, and meteorological data, the proposed IoT-enabled LWSN with EFKF can contribute to performance improvements of air quality monitoring in local areas in concert with the State-run stations. For this, machine learning techniques including artificial neural networks are promising to assemble models for the best prediction of air quality profiles [36]. Along with accuracy as one of the foremost important problems in any monitoring systems, future work could be directed to address difficulties in the LWSN deployment of the nodes, scalability, coverage, connectivity, and security.

## 6. Conclusions

This paper has reported the development of an IoT-enabled low-cost wireless sensor network for air quality monitoring in suburban areas. The system features collocated sensor motes within an ESP-NOW network, each with several sensor modules of various air pollutant sensors, WiFi connected to the server, supplied by solar panels, and is enabled by an Internet of Things microcontroller. Dependable schemes and an extended fractional-order Kalman filtering algorithm are developed and supplemented the air quality monitoring system to enhance its accuracy and reliability. After laboratorial testing of a prototype, the wireless sensor network is applied to monitor air pollutant distributions of a construction site in a suburb with sensor motes installed on-site being commercial Environmental Monitoring Systems. Particulate matters data were recorded over 6 months in association with a bushfire event, a normal session and a COVID-19 lockdown period. Local measurements are compared with data collected from the closest available State-run stations, and show good linear correlation. Field results obtained are presented and analyzed by using statistical metrics, indicating the ability to cope with imperfectness such as missing data and to provide highly-accurate estimation of dust distributions in various atmospheric events. The proposed system is promising for microclimate responsiveness. Our future work will focus on blending the existing models for air pollution forecast.

## Figures and Tables

**Table 1:** Main components of each sensor module.

Components	Features	Cost (USD)
Sensor SDS011	PM2.5 and PM10	21
BME280	Air pressure, RH and temperature	5
Soil Moisture	Corrosion resistance type	6
Kit ESP32 Node MCU	IoT Microcontroller	7
TP4506	Charging battery	0.4
HT016	DC-to-DC converter	0.4
Solar panel	Harvesting solar energy	7
Battery 18650	Supply voltage (2 cells)	12
Box (IP68)	Water and dust protection	25

**Table 2:** Performance statistics of EFKF and EKF in dataset of PM2.5 during bushfire event.

Performance Indices→	EFKF			EKF		
Nodes↓	MAE	RMSE	PCC	MAE	RMSE	PCC
EMS1	1.4752	3.4059	0.9996	3.9403	11.9035	0.9749
EMS2	1.4100	3.2470	0.9995	4.0939	11.6385	0.9731
EMS3	1.5599	3.6213	0.9995	4.5112	12.9454	0.9738
EMS4	1.5323	3.7088	0.9995	4.6182	13.5483	0.9730
EMS5	1.1516	2.1521	0.9997	2.5089	5.0527	0.9862
EMS6	1.1754	1.7911	0.9997	2.1400	3.7255	0.9865
EMS7	1.5810	3.8564	0.9995	4.6820	13.7309	0.9742
EMS8	1.5014	3.7478	0.9995	4.5629	13.2431	0.9752

**Table 3:** Performance statistics of EFKF and EKF in dataset of PM10 during normal session.

Performance Indices→	EFKF			EKF		
Nodes↓	MAE	RMSE	PCC	MAE	RMSE	PCC
EMS1	0.2069	0.2895	0.9995	0.6748	0.9410	0.9600
EMS2	0.2216	0.3311	0.9992	1.0032	1.4237	0.9362
EMS3	0.2009	0.3210	0.9995	0.8496	1.2745	0.9544
EMS4	0.2072	0.3133	0.9994	0.9974	1.4191	0.9361
EMS5	0.1766	0.2886	0.9994	0.9049	1.4601	0.9290
EMS6	0.1679	0.2755	0.9992	0.8110	1.2135	0.9410
EMS7	0.2185	0.3397	0.9994	0.9797	1.3946	0.9487
EMS8	0.2360	0.3689	0.9995	1.0202	1.4850	0.9501

**Table 4:** Performance statistics of EFKF and EKF in dataset of PM1 during COVID-19 lockdown.

Performance Indices→	EFKF			EKF		
Nodes↓	MAE	RMSE	PCC	MAE	RMSE	PCC
EMS1	0.2294	0.3920	0.9996	0.7945	1.3208	0.9698
EMS2	0.1673	0.3012	0.9997	0.5719	0.9531	0.9747
EMS3	0.1917	0.3415	0.9997	0.6505	1.0604	0.9753
EMS4	0.1790	0.3388	0.9996	0.6847	1.3128	0.9633
EMS5	0.1613	0.3064	0.9996	0.6221	1.1606	0.9653
EMS6	0.1624	0.3061	0.9997	0.6158	1.0191	0.9732
EMS7	0.1836	0.3502	0.9997	0.6178	1.0308	0.9790
EMS8	0.1789	0.3395	0.9998	0.4979	0.8402	0.9849
EMS9	0.2086	0.3600	0.9994	0.8928	1.7137	0.9408
EMS10	0.1617	0.3007	0.9996	0.6130	1.1494	0.9641
EMS11	0.2621	0.4801	0.9998	0.6013	1.0920	0.9868
EMS12	0.1828	0.3281	0.9997	0.6513	1.0972	0.9717
EMS13	0.2348	0.3962	0.9996	0.9219	1.5618	0.9586
EMS14	0.1679	0.3094	0.9996	0.6249	1.1411	0.9662
EMS15	0.2269	0.4122	0.9996	0.9183	1.7171	0.9563

## Data Availability

Not applicable.
